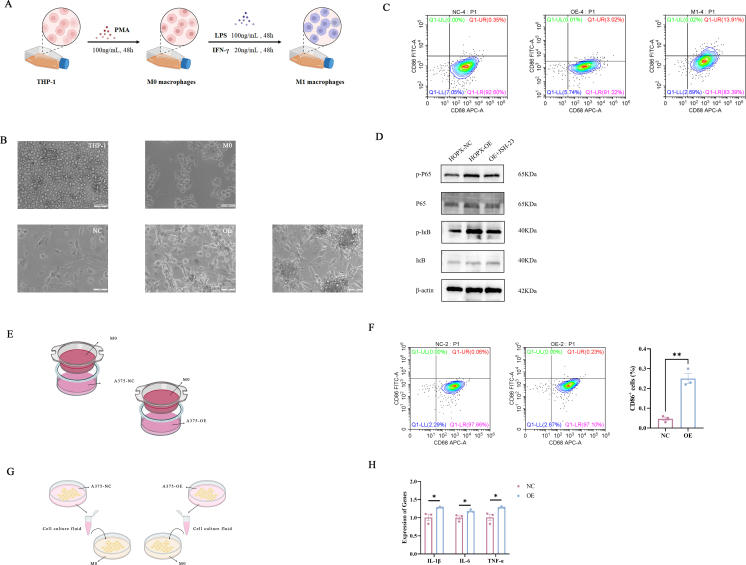# HOPX inhibits skin cutaneous melanoma growth and induces macrophage M1 polarization

**DOI:** 10.1016/j.gendis.2025.101550

**Published:** 2025-01-28

**Authors:** Xiwen Zhang, Song He, Qing Zhang, Zhonghao Ji, Jianze Zheng, Luyao Cui, Bao Yuan, Jian Chen, Yu Ding

**Affiliations:** a Department of Laboratory Animals, College of Animal Sciences, Jilin University, Changchun, Jilin 130062, China; b Frontiers Science Center for Disease-related Molecular Network, West China Hospital of Sichuan University, Chengdu, Sichuan 610097, China

Skin cutaneous melanoma (SKCM) is a malignancy arising from the transformation of melanocytes in the basal layer of the epidermis, which accounts for over 75% of skin cancer-related deaths.^1^ Homeodomain-only protein homeobox (HOPX) is the smallest member of the known homeodomain protein family in terms of relative molecular weight, and it was identified in 2002.^2^ Recently, it has been reported that HOPX can act as a tumor suppressor and is also able to participate in immune regulation,^3^^,^^4^ however, its role and mechanisms in SKCM and its modulation of immune cells remain unclear. Therefore, in this study, we aimed to investigate the role and mechanisms of HOPX on SKCM growth and its potential impact on macrophage polarization.

Research has reported that high methylation of the HOPX promoter DNA can lead to gene silencing in gastric cancer cells.^5^ Whether HOPX undergoes methylation modification in SKCM is unclear. According to the results of the UALCAN database, there was a trend towards relatively higher levels of HOPX promoter methylation in the SKCM group (primary and metastatic types) compared with the normal group (Fig. S1A). Additionally, the TCGA database results showed that the expression of DNA methyltransferase 1 (DNMT1) and DNA methyltransferase 3 alpha (DNMT3A) was significantly elevated in SKCM (*P* < 0.001) (Fig. S1B, C), suggesting potential methylation modifications of HOPX in SKCM. Subsequently, SKCM cell lines A375 and A875 were treated with decitabine (a DNA methyltransferase inhibitor) at a concentration of 10 μM, and surprisingly, both HOPX gene and protein levels were significantly increased (Fig. 1A–C; Fig. S1J–L). This implies a close association between the low expression of HOPX in SKCM and its methylation modification. Furthermore, an in-depth investigation of the promoter methylation modification sites of HOPX indicated methylation alterations upstream of the HOPX transcription start site within the range of 300–800 bp (*P* ≤ 0.001). Specifically, the sites were identified as cg04085076 (TSS+487), cg25456368 (TSS+719), cg00019495 (TSS+541), and cg06771126 (TSS+367) (Fig. S1D–I).

**Figure 1:**
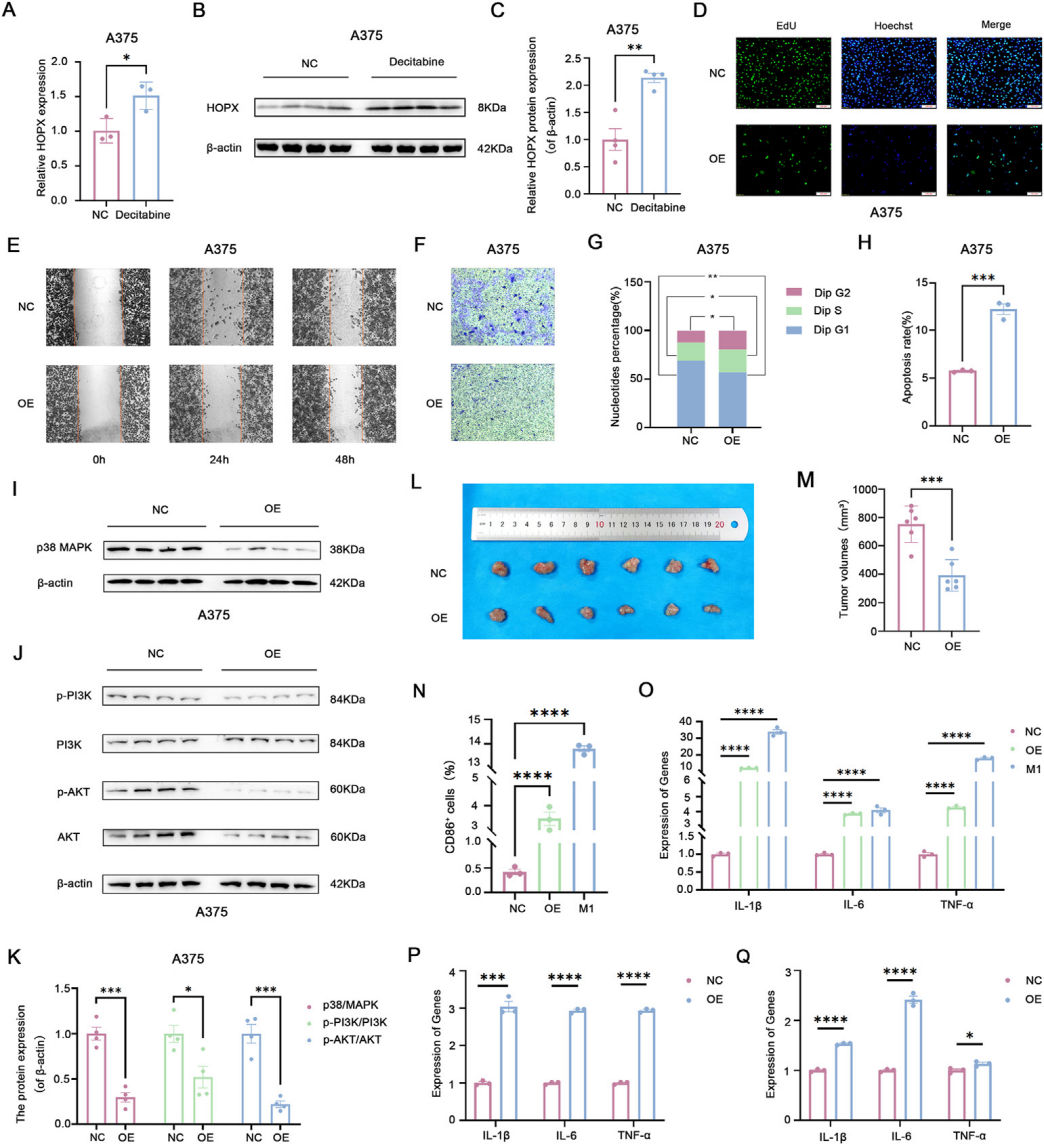
HOPX is hypermethylated and modified in skin cutaneous melanoma (SKCM) and it inhibits cell growth and induces macrophage M1 polarization. **(A)** Decitabine treatment of A375 cells increased the gene expression levels of HOPX. **(B, C)** Decitabine treatment of A375 cells increased the protein expression levels of HOPX. **(D)** EdU staining assay was used to detect HOPX inhibition of A375 cell proliferation. **(E)** The wound healing assay was used to detect the ability of HOPX to inhibit A375 cell migration. **(F)** Transwell with Matrigel assay was used to detect the ability of HOPX to inhibit A375 cell invasion. **(G)** Flow cytometric detection of HOPX promotes S-phase arrest in A375 cells. **(H)** HOPX promotes apoptosis in A375 cells as detected by flow cytometry. **(I–K)** HOPX decreases the expression level of p38 MAPK protein as well as PI3K & Akt phosphorylated proteins in A375 cells. **(L)** Subcutaneous tumor excision in control (NC) and experimental (OE) nude mice (*n* = 6). **(M)** Tumor volumes excised from experimental (OE) nude mice were significantly smaller than control (NC). **(N)** Flow cytometry detection of HOPX-induced macrophage M1 polarization. **(O)** Quantitative reverse transcription PCR detection of HOPX increased expression levels of inflammatory factors in macrophages. **(P)** Effect of transwell co-culture method on cellular inflammatory factors detected by quantitative reverse transcription PCR. **(Q)** Effect of conditioned medium (the ratio of A375 cell culture medium to M0 macrophage cell culture medium was 1:1) co-culture method on cellular inflammatory factors detected by quantitative reverse transcription PCR. ns, *P* ≥ 0.05; ∗*P* < 0.05, ∗∗*P* < 0.01, ∗∗∗*P* < 0.001, and ∗∗∗∗*P* < 0.0001.

Previous studies have found that HOPX enhances sensitivity to chemotherapeutic agents in SKCM patients.^3^ We explored this using cisplatin and tamoxifen in predicted outcomes in A375 and A875 cells. It was found that HOPX reduced the IC50 of cisplatin and tamoxifen in SKCM cell lines and increased the sensitivity of chemotherapeutic agents in SKCM (Fig. S2), which also provides insights into the application of precision medicine and anti-drug resistance in clinical SKCM patients.

We further investigated the effect of HOPX on SKCM cell lines using A375 cells and A875 cells. The CCK-8 assay showed that HOPX was able to reduce the viability of SKCM cells (Fig. S3A, G), as aided by EdU staining and colony-formation assays (Fig. 1D; Fig. S3B, H, M). Furthermore, the wound healing and transwell assays suggested a significant reduction in the migration capability of SKCM cells (Fig. 1E; Fig. S3C–E, I–K, N). Transwell with Matrigel results similarly indicated that HOPX significantly lowered the invasion capability of cells (Fig. 1F; Fig. S3F, L, O). Preliminary results found a positive regulatory relationship between HOPX expression and apoptosis but negatively correlated with the citrate cycle^3^ (Fig. S4). Further, flow cytometry results showed that HOPX significantly induced S-phase arrest and increased the proportion of apoptotic cells in SKCM cells (Fig. 1G, H; Fig. S5A–C, H). Western blot results also showed that the expression levels of cleaved caspase3 and Bax proteins were significantly increased in A375 and A875 cells after HOPX overexpression treatment, but Bcl2 proteins were not (Fig. S5D–G, I–L).

The results of transcriptome sequencing showed that the involvement of HOPX in the regulation of SKCM may be related to the p38 MAPK (mitogen-activated protein kinase) and PI3K (phosphoinositide 3-kinase)-Akt (protein kinase B) signaling pathways, so western blotting was used to detect the expression levels of the relevant proteins. The results showed that p38 MAPK protein expression levels were significantly reduced in A375 and A875 cells after HOPX overexpression treatment (Fig. I, K; Fig. S6A), and protein levels of p-PI3K/PI3K and p-Akt/Akt were also significantly reduced (Fig. 1J, K; Fig. S6B). These suggest that HOPX may be involved in SKCM cell development through the p38 MAPK/PI3K-Akt signaling pathway.

The effect of HOPX on SKCM cell growth is evaluated *in vivo*, using a nude mouse model of human SKCM cell lines. In particular, A375 cells stably expressing HOPX are injected under the axillary skin of nude mice (Fig. S7A). The result showed that the tumor volume and weight were significantly reduced in the HOPX overexpression group compared with the control group (Fig. S7B, C). The results indicated that HOPX was able to inhibit the growth of SKCM cells *in vivo* (Fig. 1L, M; Fig. S7D, E).

Macrophages are immune effector cells that also perform anti-tumor or protumor functions in different settings. Preliminary findings from our group suggest that HOPX regulation of SKCM involves immune infiltration and is closely linked to macrophage^3^ (Fig. S8). Then an effect of HOPX on macrophage polarization was explored using THP-1 cells (Fig. S9A). The morphology of THP-1 cells changed significantly after treatment with phorbol-12-myristate-13-acetate, with cells changing from suspended spherical to walled irregular, and further increasing in cell volume, with pronounced nuclei and increased cytoplasm (Fig. S9B). Flow cytometry results showed that HOPX could significantly increase the proportion of CD86^+^ positive cells (Fig. 1N; Fig. S9C). By the way, we further explored the underlying molecular mechanism and found that HOPX promoted phosphorylation of IкB (inhibitory kappa B) and P65, which was reversed by the inhibitor JSH-23 (MCE, HY13982) (Fig. S9D). In addition, quantitative reverse transcription PCR results showed that HOPX could significantly increase the expression levels of pro-inflammatory cytokines such as interleukin-1β (IL-1β), interleukin-6 (IL-6), and tumor necrosis factor-alpha (TNF-α) (Fig. 1O).

Furthermore, A375 cell and M0 macrophage co-culture was also utilized and performed using the transwell chamber and the conditioned medium co-culture method (Fig. S9E, G). Transwell chamber co-culture results showed that HOPX was able to increase the number of CD86^+^ positive cells and inflammatory factor expression levels (Fig. 1P; Fig. S9F). The results of the co-culture of the conditioned medium showed that HOPX could increase the expression level of inflammatory cytokines, and the effect was more pronounced with a higher proportion of cancer cell cultures in the co-culture (Fig. 1Q; Fig. S9H). The results of the study showed that HOPX was able to induce macrophage M1 polarization and increase the expression of inflammatory cytokines.

To conclude, this research demonstrates the close association of low expression of HOPX with methylation modifications in SKCM. HOPX inhibits SKCM cell growth, promotes apoptosis and S-phase arrest, and inhibits activation of the p38 MAPK/PI3K-Akt signaling pathway. Importantly, it also reduces the resistance to clinical chemotherapeutic drugs, promotes M1 polarization of macrophages, and participates in immune therapy for tumors. Considering prior foundational studies and the outcomes of this research, HOPX is expected to be able to become a new target for clinical treatment in SKCM, providing novel strategies for innovative immunotherapy in clinical settings.

## Ethics declaration

The experimental procedure was conducted in strict compliance with the Care and Use of Laboratory Animals of Jilin University. The experimental protocol was approved by the Animal Welfare Ethics Committee of Jilin University (No. SY202309006), and the animal experiments were carried out at the Jilin University Laboratory Animals Centre.

## Funding

This study is supported by the National Key Research and Development (R&D) Program of China (No. 2022YFF0710503, 2023YFF0724600), the Jilin Provincial Science and Technology Development Plan Project (China) (No. 20230505037ZP), and the Graduate Innovation Fund of 10.13039/501100004032Jilin University (China) (No. 2024CX290).

## CRediT authorship contribution statement

**Xiwen Zhang:** Writing – original draft, Validation, Methodology, Investigation, Data curation, Conceptualization. **Song He:** Writing – original draft, Software, Methodology, Investigation, Data curation, Conceptualization. **Qing Zhang:** Visualization, Validation, Supervision, Conceptualization. **Zhonghao Ji:** Validation, Supervision, Software, Formal analysis. **Jianze Zheng:** Methodology, Investigation. **Luyao Cui:** Methodology, Investigation. **Bao Yuan:** Supervision, Resources, Project administration. **Jian Chen:** Writing – review & editing, Validation, Supervision, Project administration. **Yu Ding:** Writing – review & editing, Validation, Supervision, Project administration, Funding acquisition.

## Data availability

In this study, the transcriptome sequencing results performed have been uploaded into the public database GEO and are publicly available (record number GSE221101).

## Conflict of interests

The authors declared no conflicting interest in the authorship and publication of this study.

## Figures and Tables

**figs1:**
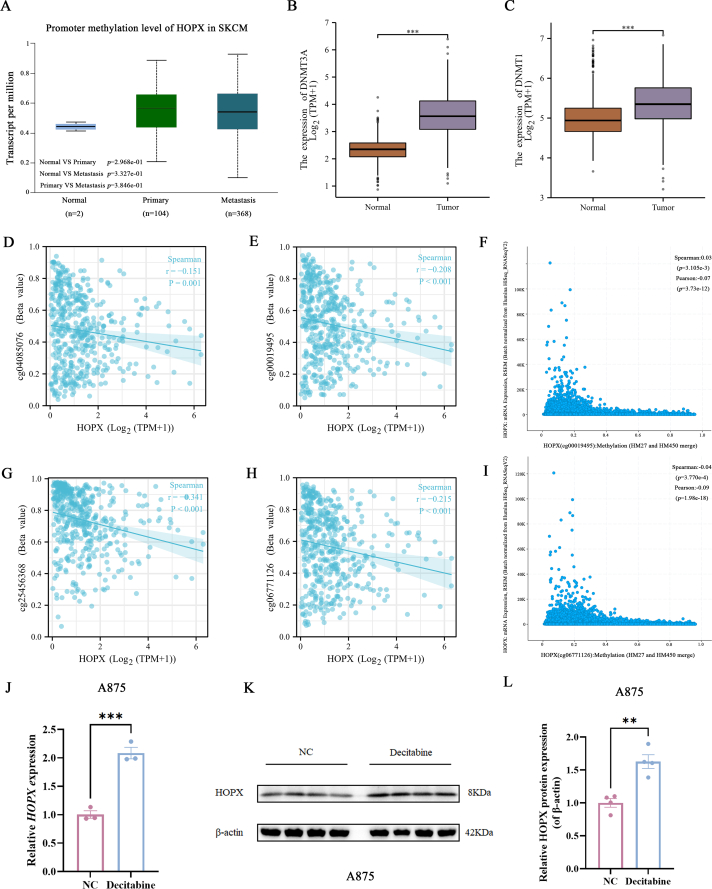


**figs2:**
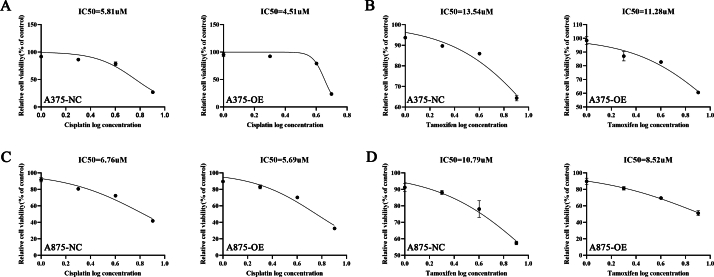


**figs3:**
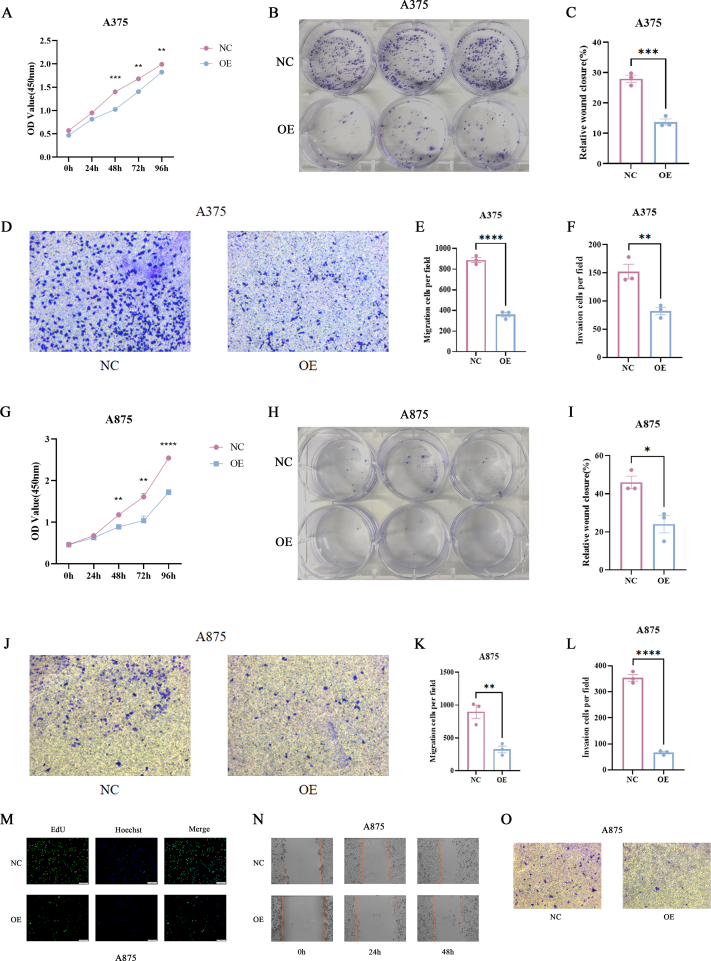


**figs4:**
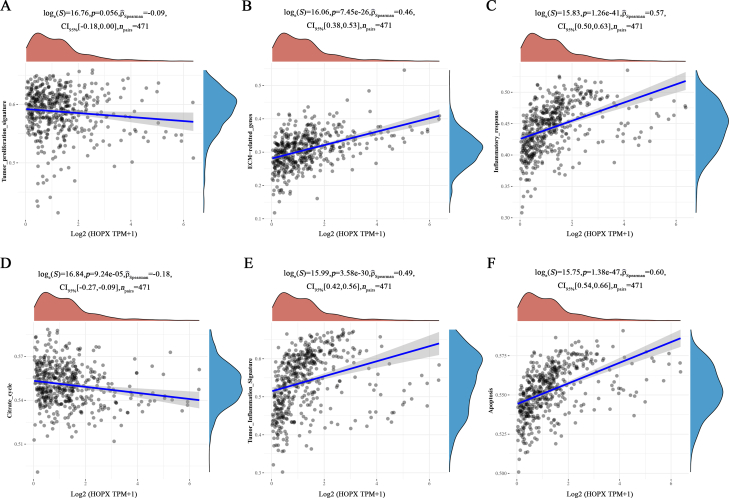


**figs5:**
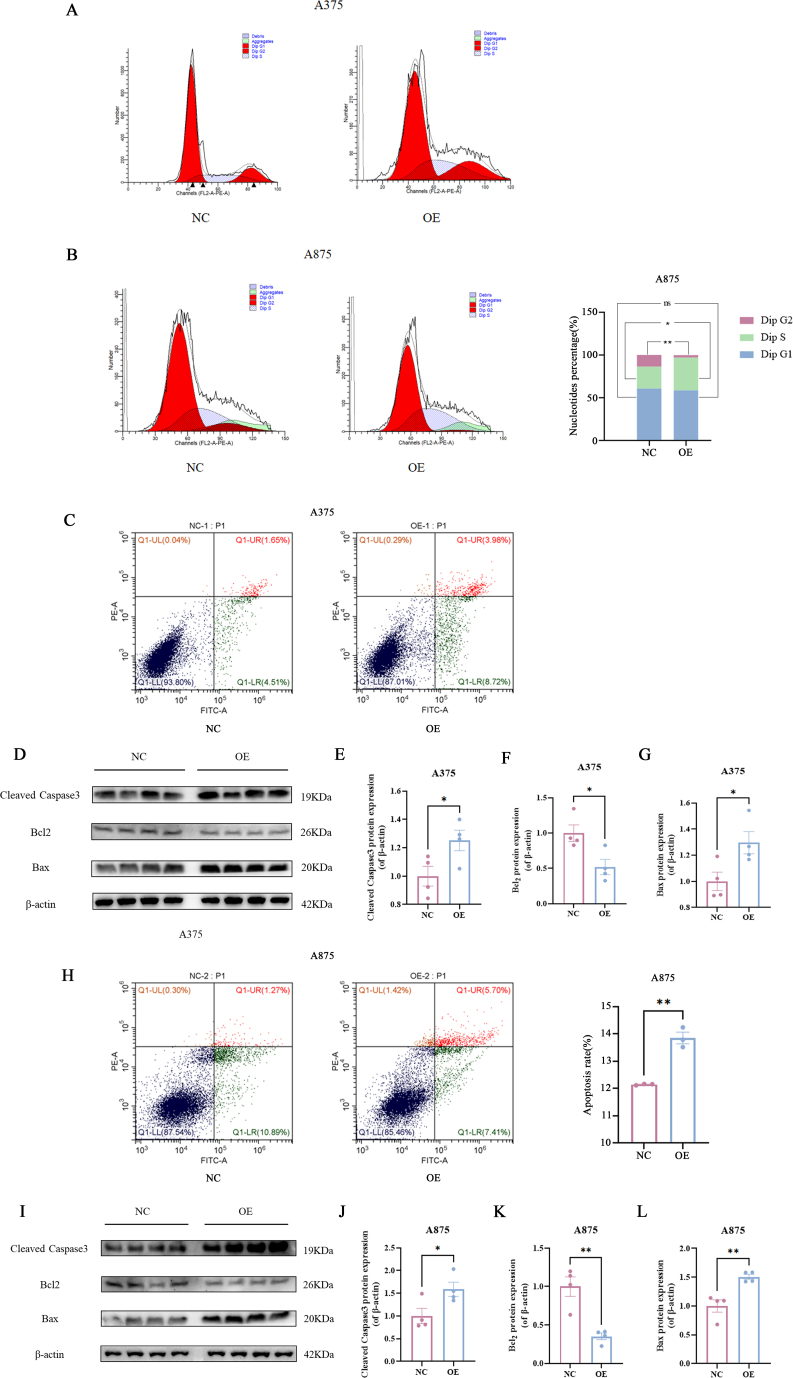


**figs6:**
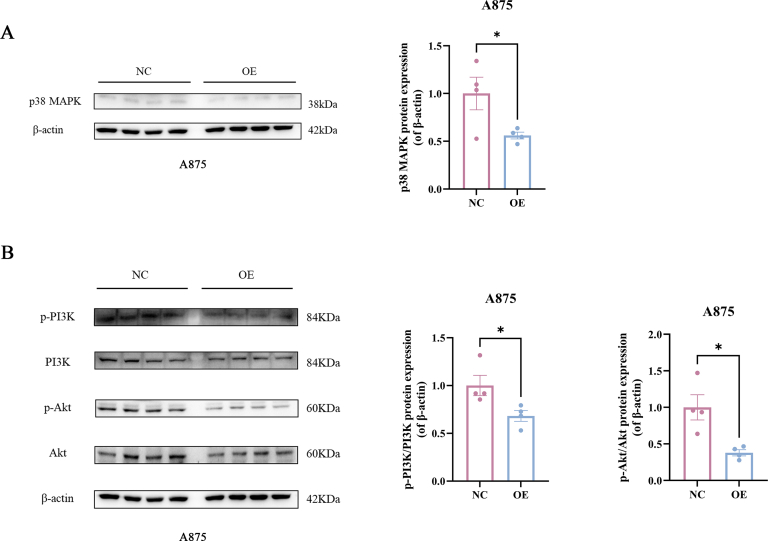


**figs7:**
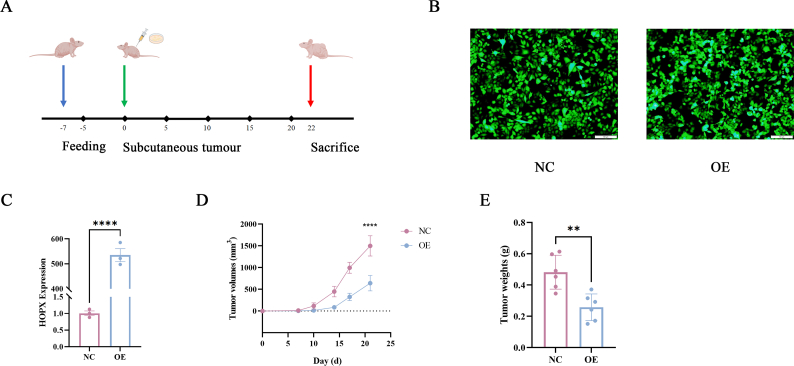


**figs8:**
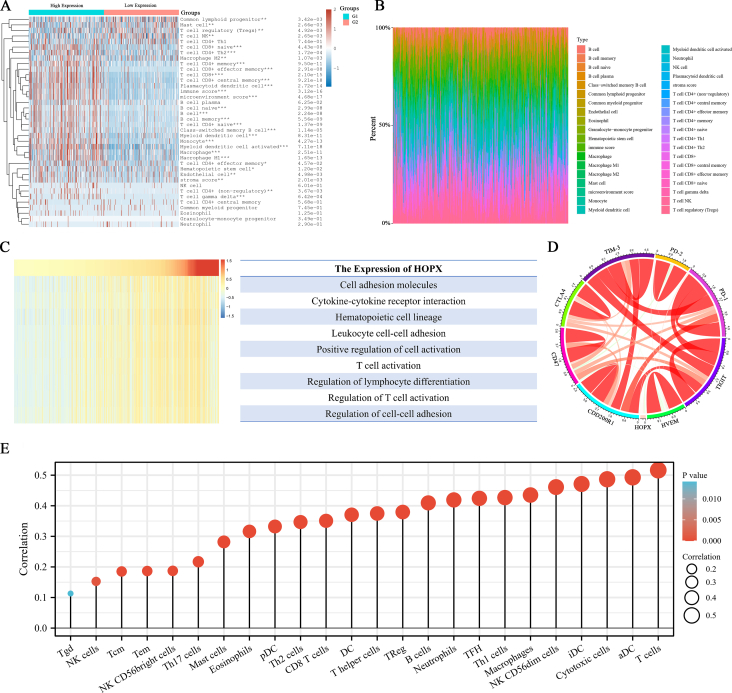


**figs9:**