# Supplementary material for: HOPX inhibits skin cutaneous melanoma growth and induces macrophage M1 polarization
Source: Genes Dis. 2025 Jan 28;12(5):101550. doi: 10.1016/j.gendis.2025.101550 (PMC12166724; doi:10.1016/j.gendis.2025.101550)
Supplement: Multimedia component 1 [file mmc1.docx]

**Materials and Methods**

**Cell culture**

Human malignant skin cutaneous melanoma cell lines A375 and A875 were cultured in DMEM (Sigma, USA) and MEM (Sigma, USA), respectively, supplemented with 10% fetal bovine serum (FBS) (Lonsera, UY) and 1% penicillin-streptomycin (P/S) solution (Beyotime, CHN). Human mononuclear leukaemia THP-1 cells were cultured in RPMI-1640 (MeilunBio, CHN) containing 10% FBS, 1% P/S, and 0.05 mM 2-mercaptoethanol (Aladdin, USA). All cells were maintained at 37°C in a humidified incubator (SANYO, JPN) with 5% CO_2_. A375 cells were obtained from Boster Biotech (Wuhan, CHN), A875 cells from ZQXZ Biotech (Shanghai, CHN), and THP-1 cells from MeilunBio (Dalian, CHN).

**Animal ethics and cell line-derived xenograft (CDX) models**

BALB/c-nude mice (5-7 weeks old) were obtained from Jiangsu Huachuang Xinnuo Pharmaceutical Technology Co. (Jiangsu, CHN). The experimental protocol was approved by the Animal Welfare Ethics Committee of Jilin University (Grant No. SY202309006). All animals were free to drink and feed, and all experimental procedures were conducted in strict compliance with animal welfare ethics and animal welfare laws and regulations.

All the animals were randomly divided into 2 groups, namely NC group and OE group, with 6 nude mice in each group (3 males and 3 females), and subcutaneous xenograft tumor models were established using A375 cells. When appropriate, cells were collected and suspended in 1 mL sterile PBS, so that each nude mouse was injected subcutaneously with 150 μL containing 5.0×10^6^ cells. When the tumor size reached 1500 mm^3^, the nude mice were euthanized, the tumors were excised, and statistical analysis was performed. Tumor volume = (width)^2^ × length /2.

**Transfection and stable cell line construction**

HOPX overexpression plasmid and lentiviral vectors were synthesized by Sangon Biotech (Shanghai, CHN), and the HOPX overexpression SKCM cells were constructed according to the manufacturer's instructions. Additionally, Puromycin (5 μg/mL) was used for screening stable HOPX-expressing A375 cells.

**RNA extraction, cDNA synthesis and quantitative real-time PCR (RT‒qPCR)**

RNA was extracted from cells, and cDNA synthesis was performed using reverse transcription according to the instructions. RT-qPCR was conducted using ChemoHS qPCR Mix (Monad, CHN) and Eppendorf Quantitative PCR Instrument (Eppendorf, CHN).

**Cell viability assays**

Resuspension A375 cells or A875 cells and seed them in 96-well plates (5000 cells per well). After cell transfection or drug treatment is completed, 10 μL of CCK-8 reagent (MCE, USA) was added to every 100 μL of cell culture medium. After 1.5 hours, the optical density (OD) value at 450 nm was measured using a microplate reader (TECAN, AUT).

**EdU staining assays**

Resuspend A375 or A875 cells and seed them in a 6-well plate. After the appropriate number of cells, the cells were labelled with Meilun EdU Cell Proliferation Kit with Alexa Fluor 488 Kit (MeilunBio, CHN) according to the instructions and incubated with DAPI. Finally, detection was performed using fluorescence microscopy.

**Colony-formation assays**

The transfected A375 or A875 cells are resuspended and counted, then grown in 6-well plates (1000 cells per well) and the medium is changed when the cells are stable. When visible colonies were formed, the medium was discarded and the cells were fixed with 4% paraformaldehyde and stained with 0.1% crystal violet. Afterwards images were acquired and analyzed.

**Wound healing assays**

Resuspend A375 or A875 cells and seed them in a 6-well plate, when the cell density is appropriate, use a 200 µL pipette tip was used to form a scratch on the monolayer of cells, which was then rinsed three times with PBS to remove cellular debris, etc. The cells continued to be cultured using serum-free medium and the wound site was imaged at the detection time point and then analyzed.

**Transwell migration and invasion assays**

Transfected A375 or A875 cells were resuspended and counted, grown in the upper layer of Transwell chambers and continued in serum-free medium. In contrast, invasion experiments were performed by first adding an appropriate amount of Matrigel (ABW, CHN) in the upper part of the Transwell. The cells were fixed with 4% paraformaldehyde and stained with 0.1% crystal violet and analyzed under an inverted microscope.

**Cell cycle and apoptosis assays**

After transfection of A375 or A875 cells, the cells were treated with the Cell Cycle and Apoptosis Analysis Kit (Beyotime, CHN) according to the instructions, after which the cell cycle and apoptosis were detected by flow cytometry (Beckman, USA).

**Western blotting**

After transfection or treatment of A375 or A875 cells was completed, proteins were extracted using RIPA lysis buffer (Epizyme, CHN) and protein content was quantified according to the BCA Protein Assay Kit (Beyotime, CHN). SDS-PAGE was performed to separate each sample and transferred to PVDF membranes (Immobilon-P, IRL). After these blots were blocked with Protein Frenn Rapid Blocking Buffer (Epizyme, CHN), the membranes were incubated with the appropriate primary antibody and secondary antibodies. Finally, protein levels were quantified using ECL Chemiluminescence Kit (Beyotime, CHN) using an automatic chemiluminescence/fluorescence image analysis system (Tanon 5200, CHN). The antibodies used in this experiment were anti-HOPX (1:500, #11419-1-AP, Proteintech), anti-β-actin (1:1000, #4967S, CST), anti- p-PI3K (1:1000, #AF3241, Affinity), anti-PI3K (1:1000, #AF6241, Affinity), anti-p-Akt (1:1000, # BS4006, Bioworlde), anti-Akt (1:1000, #BS1811, Bioworlde), anti-p38 MAPK (1:1000, #A5017, Selleck), anti-cleaved caspase3 (1:1000, #19677-1-AP, Proteintech), anti-Bcl2 (1:1000, #AF6139, Affinity), anti-Bax (1:1000, #AF0120, Affinity) , anti-P65 (1:1000, #AF5006, Affinity), anti-p-P65 (1:1000, #AF2006, Affinity), anti-IкB (1:1000, #AF5002, Affinity), anti-p-IкB (1:1000, AF2002, Affinity),and anti-rabbit IgG (H+L)-HRP (1:2000, #A0208, Beyotime).

**Macrophage polarization assays**

After centrifuging and resuspending, THP-1 cells were seeded in a 6-well plate. Treating these cells with PMA (100 ng/mL, MCE, USA) for 48 h can induce their differentiation into M0 macrophages. Subsequently, intervention with LPS (100 ng/mL, MCE, USA) and IFN-γ (20 ng/mL, PeproTech, USA) for another 48 h can further induce their differentiation into M1 macrophages. When the processed cells were labelled with CD68 and CD86 (4A Biotech, CHN), they were detected and analyzed using flow cytometry.

**Cell co-culture assays**

The transfected A375 cell culture medium was collected and added to the 6-well plates cultured with M0 macrophages at a ratio of 1:1 or 1:2 to continue the culture (that is, the A375 cell culture medium accounted for 1/2 or 1/3 of the total culture medium), after which the expression levels of relevant inflammatory factors were detected.

The A375 cells were resuspended and seeded into a 6-well plate. Upon completion of transfection, Transwell chambers were placed into the 6-well plate, and M0 macrophages were seeded in the upper chamber. Subsequently, macrophage polarization and the expression levels of relevant inflammatory factors were assessed.

**Illumina sequencing**

Single-end sequencing was performed on the Illumina Novaseq^TM^ 6000 platform provided by LC Biotech (Hangzhou, CHN) and the sequencing results were deposited in the GEO database (NO. GSE221101).

**Correlation analysis of methylation expression**

In the TCGA dataset (https://portal.gdc.cancer.gov/), we downloaded RNA-sequencing expression (level 3) profiles and illumina human methylation 450 states related to SKCM and validated with the aid of the cBioPortal database (https://www.cbioportal.org/) and the UALCAN database (https://ualcan.path.uab.edu/index.html). The data were fully analyzed using the ggplot2 package in R.

A375 or A875 cells were resuspended and grown in 6-well plates, and when the cell density was appropriate, Decitabine (10 μM, MCE, USA) was added for treatment, and finally RNA and protein were extracted to detect the expression level of HOPX.

**Statistical analyses**

All data were presented as mean ± SD from three independent biological replicates. Statistical significance was determined using Student's t-tests and one-way ANOVA, with *P*<0.05 considered significant (ns, *P*≥0.05; **P*<0.05, ***P*<0.01, ****P*<0.001, *****P*<0.0001).

**Supplementary Legends**

**Figure S1.** Methylation-related analysis of HOPX in SKCM. **(A)** Promoter methylation levels of HOPX in SKCM from the UALCAN database. **(B, C)** Expression levels of DNA methyltransferases (DNMT3A and DNMT1) in SKCM from TCGA database. **(D-G)** TCGA database of methylation probes associated with HOPX in SKCM. Specifically, the sites were identified as cg04085076 (TSS+487), cg25456368 (TSS+719), cg00019495 (TSS+541), and cg06771126 (TSS+367). **(H, I)** The cBioPortal database of methylation probes linked to HOPX in SKCM. **(J)** Treatment of decitabine with A875 cell increased the gene expression levels of HOPX. (**K, L)** Treatment of decitabine with A875 cell increased the protein expression levels of HOPX.

**Figure S2.** HOPX reduces resistance to clinical chemotherapy drugs. **(A)** HOPX decreases IC50 of cisplatin in A375 cells and **(C)** A875 cells. **(B)** HOPX decreases IC50 of tamoxifen in A375 cells and **(D)** A875 cells.

**Figure S3.** HOPX reduces the viability of A375 and A875 cells and inhibits their cell proliferation, migration, and invasion. **(A)** The CCK-8 assay was used to detect the reduction of A375 cell viability by HOPX. **(B)** Colony formation assay was used to detect HOPX inhibition of A375 cell clone-formation. **(C)** The wound healing assay showed that HOPX inhibited A375 migration. **(D, E)** The Transwell assay was used to detect the ability of HOPX to inhibit A375 migration. **(F)** The Transwell with Matrigel assay showed that HOPX inhibited A375 invasion. **(G)** The CCK-8 assay was used to detect the reduction of A875 cell viability by HOPX. **(H)** Colony formation assay was used to detect HOPX inhibition of A875 cell clone-formation. **(I)** The wound healing assay showed that HOPX inhibited A875 migration. **(J, K)** The Transwell assay was used to detect the ability of HOPX to inhibit A875 migration. **(L)** The Transwell with Matrigel assay showed that HOPX inhibited A875 invasion. **(M)** EdU staining assay was used to detect HOPX inhibition of A875 cell proliferation. **(N)** The wound healing assay was used to detect the ability of HOPX to inhibit A875 cell migration. **(O)** Transwell with Matrigel assay was used to detect the ability of HOPX to inhibit A875 cell invasion.

**Figure S4.** Evaluation of the relationship between HOPX and tumor progression. **(A)** HOPX was negatively correlated with tumor proliferation and **(D)** the citrate cycle. **(B)** HOPX was positively correlated with ECM-related genes, **(C)** inflammatory response, **(E)** tumor inflammation signature **(F)** and apoptosis.

**Figure S5.** HOPX promotes cell apoptosis and S-phase arrest in A375 and A875 cells. **(A, B)** Flow cytometric detection of HOPX promotes S-phase arrest in A375 and A875 cells. **(C, H)** HOPX promotes apoptosis in A375 and A875 cells as detected by flow cytometry. **(D-G, I-L)** HOPX promoted increased expression of pro-apoptotic proteins (Cleaved Caspase 3 & Bax) and decreased expression of anti-apoptotic proteins (Bcl2) in A375 and A875 cells as detected by Western Blot.

**Figure S6.** HOPX inhibits the activation of p38 MAPK/PI3K-Akt signaling pathway in A875 cell. **(A)** HOPX decreases p38 MAPK protein expression levels in A875 cell. **(B)** HOPX decreased the phosphorylated expression levels of PI3K & Akt proteins in A875 cell.

**Figure S7.** HOPX inhibits A375 cell growth in nude mice. **(A)** Pattern diagram for cell derived xenograft (CDX) model construction. **(B)** Fluorescence microscopy was used to detect the construction of stable cell lines. **(C)** RT-qPCR was performed to detect the high expression of HOPX in A375 cell. **(D)** Changes in tumor volume of CDX model at different time points. **(E)** Tumor weights excised from nude mice in the HOPX high-expression were significantly smaller than controls.

**Figure S8.** Correlation analysis between HOPX and immune function. **(A)** HOPX immunoscore heatmap of immune cell scores with high and low expression in SKCM tissue and **(B)** percentage abundance of infiltrating immune cells for each sample. **(C)** Heatmap of HOPX and immune function enrichment scores. **(D)** Pearson correlation between HOPX and immune checkpoints. **(E)** Analysis of the correlation between HOPX and immune cells.

**Figure S9.** HOPX induces macrophage M1 polarization and increases inflammatory factors expression. **(A)** Pattern diagram of macrophage polarization induction. **(B)** The morphological stages of macrophages under the light microscope field of view. **(C)** Flow cytometry detection of HOPX-induced macrophage M1 polarization.**(D)** The expression of β-actin,P65,p-P65,IкB and p-IкB proteins was detected using WB assay. **(E)** Pattern diagram of Transwell cell co-culture. **(F)** Flow cytometry detection of HOPX-induced macrophage M1 polarization. **(G)** Pattern diagram of conditioned media cell co-culture. **(H)** Effect of conditioned media (The ratio of A375 cell culture medium to M0 macrophage cell culture medium is 1:2) co-culture method on cellular inflammatory factors.
